# Implementation of goal-directed fluid therapy during hip revision arthroplasty: a matched cohort study

**DOI:** 10.1186/s13741-016-0056-x

**Published:** 2016-12-13

**Authors:** Marit Habicher, Felix Balzer, Viktor Mezger, Jennifer Niclas, Michael Müller, Carsten Perka, Michael Krämer, Michael Sander

**Affiliations:** 1 Department of Anaesthesiology and Intensive Care Medicine, Charité University Hospital Berlin, Campus Charité Mitte and Campus Virchow-Klinikum, Berlin, Germany; 2 Centre for Musculoskeletal Surgery, Department of Orthopaedics, Charité University Hospital Berlin, Campus Charité Mitte and Campus Virchow-Klinikum, Berlin, Germany; 3 Department of Anaesthesiology, Intensive Care Medicine and Pain Therapy, Justus-Liebig-University, Giessen, Germany

**Keywords:** Goal-directed fluid therapy, Hip surgery, Postoperative outcome, Haemodynamic monitoring, Hip revision arthroplasty

## Abstract

**Background:**

Several randomized controlled trials (RCTs) have demonstrated that intraoperative goal-directed fluid therapy (GDFT) can decrease postsurgical complications in patients undergoing major abdominal surgery. However, very few studies have demonstrated the value of goal-directed therapy (GDT) in patients undergoing orthopaedic surgery and confirmed it is as useful in real-life conditions. Therefore, we initiated a GDFT implementation programme in patients undergoing hip revision arthroplasty in order to assess its effects on postoperative complications (e.g. infection, cardiac, neurological, renal) (primary outcome) and hospital and intensive care unit (ICU) length of stay (secondary outcomes).

**Methods:**

We developed a GDFT protocol for the haemodynamic management of patients undergoing hip revision arthroplasty. The GDFT protocol was based on continuous monitoring and optimization of stroke volume during the surgical procedure. From December 2012 and for a period of 17 months, 130 patients were treated according to the GDFT protocol (GDFT group). The pre-, intra-, and postoperative characteristics of patients from the GDFT group were compared to those of 130 historical matched patients (control group) who had the same surgery between January 2011 and August 2012.

**Results:**

Patients from the GDFT and from the control group were comparable in terms of age, comorbidities, and P-POSSUM score. Duration of anaesthesia and surgery were also comparable. The GDFT group had a significantly lower morbidity rate (49.2 vs. 66.9%; *p* = 0.006) and a shorter median hospital length of stay (11 days (9–15) vs. 9 days (8–12); *p* = 0.003) than the control group. Patients from the control group post-anaesthesia care unit (PACU)/ICU stayed significantly longer at PACU/ICU than patients from the GDFT group (control group vs. GDFT group, 960 min (360–1210) vs. 400 min (207–825); *p* < 0.001) Patients from the GDFT group received less crystalloids but more colloids during surgery. They also received more often inotropic therapy.

**Conclusions:**

In patients undergoing hip revision arthroplasty, the implementation of GDT as a new standard operating procedure was successful and associated with reduced postsurgical complications, most importantly a reduction in postoperative bleeding as well as hospital and ICU stay.

**Trial registration:**

ClinicalTrials.gov, NCT01753050

**Electronic supplementary material:**

The online version of this article (doi:10.1186/s13741-016-0056-x) contains supplementary material, which is available to authorized users.

## Background

More than 230 million major surgical procedures are undertaken every year worldwide (Weiser et al. 2008). The most operations were performed under general anaesthesia. A survey in 2013 in the UK showed over 2,766,600 general anaesthesia during 1 year (Sury et al. 2014). Morbidity rates >25% have repeatedly been reported after major surgery (Ghaferi et al. 2009). Therefore, strategies to improve outcome and prevent postoperative complications are required in surgical patients.

Many randomized controlled trials (RCTs) and meta-analysis suggest that perioperative goal-directed fluid therapy (GDFT) decreases postsurgical complications and length of hospital stay in patients undergoing major abdominal procedures (Benes et al. 2010; Gan et al. 2002; Grocott et al. 2012; Hamilton et al. 2011; Lopes et al. 2007). However, (1) larger and multicentre studies have yielded conflicting results (Pearse et al. 2014; Pestaña et al. 2014; Scheeren et al. 2013). Pearse et al. demonstrated in their randomized trial of high-risk patients undergoing major gastrointestinal surgery that the use of a cardiac output-guided haemodynamic therapy algorithm when compared did not significantly reduce postoperative complications and 30-day mortality (Pearse et al. 2014); (2) RCTs are done in highly selected patients with extra human and financial resources, such that the extrapolation of their results to the real world may be questioned (Vincent 2009); and (3) only a few studies have been done in orthopaedic patients and none in patients undergoing hip revision arthroplasty. Patients undergoing revision hip surgery are usually old and often have comorbidities, increasing their risk of complications after surgery.

Therefore, we made the decision to implement GDFT in patients undergoing hip revision arthroplasty and to assess its effects on postoperative outcome as an enhanced recovery project for these patients.

## Methods

### Study outline

We enrolled prospectively 130 patients over a period of 17 months (from December 1, 2012, to April 30, 2014) who were managed according to our GDFT protocol (GDFT group). All consecutive patients admitted for revision hip surgery were screened for inclusion. Inclusion criteria were age ≥18 years and one of the following surgical procedures (hip revision arthroplasty): hip revision with change of the prosthesis, explantation of existing hip arthroplasty, or patients after Girdlestone resection arthroplasty, who underwent new implantation of hip prosthesis. Patients from the GDFT group were compared to 130 historical matched control patients (control group) who underwent the same surgical procedure from January 1, 2011, to August 30, 2012, before we developed the algorithm for the prospective group. During this time frame, no patients were treated with additional monitoring that could measure intraoperative stroke volume.

The study protocol was registered at ClinicalTrials.gov (NCT01753050) and approved by the ethics committee at Charité – Universitätsmedizin Berlin (EA1/315/12). Informed written consent was obtained from all prospective GDFT patients. Retrospective patients (control group) provided their consent to use their data in anonymized fashion for scientific purposes by signing the treatment contract with our university hospital. The study was performed at the Charité – University hospital Berlin, Campus Charité Mitte. Stroke volume monitors were loaned by Edwards Lifesciences, which had no role in the development of the study protocol and the data analysis.

### Patient management

All patients underwent general anaesthesia during surgery. Anaesthesia was induced according to our written SOP with fentanyl (1–2 μg kg^−1^), propofol (1–2 mg kg^−1^), and cisatracurium (0.15 mg kg^−1^). After endotracheal intubation, the maintenance of anaesthesia was performed at the discretion of the attending anaesthesiologist with either sevoflurane or propofol continuously. Fentanyl and cisatracurium boli were given as needed.

**Fig. 1:**
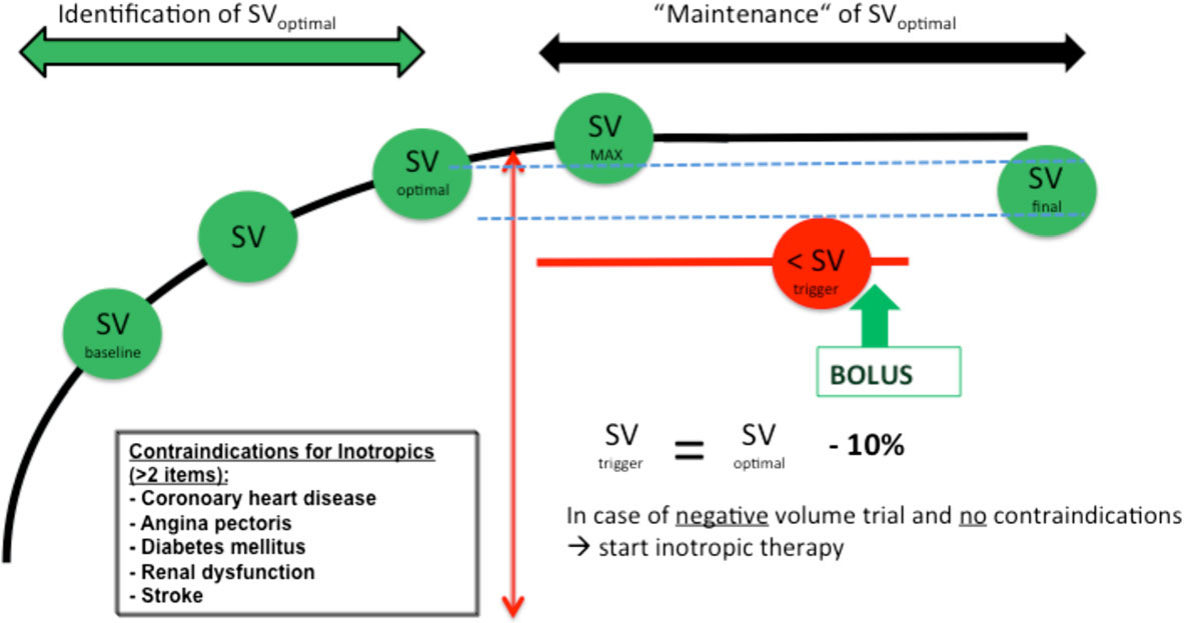
Graphical representation of our GDFT protocol. Fluid was administered until stroke volume reached a plateau value (SV_max_). The optimum SV (SV_opt_) value was the last value preceding SV_max_. SV trigger (SV_trigger_) was calculated as SV_opt_ minus 10%. Additional colloid boluses were administered only when SV was below SV_trigger_

Standard monitoring in both groups included electrocardiogram, pulse oximetry, temperature, and inspiratory and expiratory gas concentrations as well as monitoring of depth of anaesthesia. In the retrospective group, the choice between non-invasive or invasive blood pressure measurement was at the discretion of the attending anaesthesiologist. None of the patients from the retrospective group was monitored with a device that was able to measure stroke volume (SV) or any other flow-related parameter. In all patients of the GDFT group, invasive blood pressure was monitored via right or left radial artery. Haemodynamic optimization in this group was done as follows: SV was monitored using a pulse contour method (Vigileo 03.06, Edwards Lifesciences, Irvine, CA, USA) and a special pressure transducer (FloTrac system, Edwards Lifesciences). Baseline SV was measured after induction of anaesthesia and patient positioning. An intravenous colloid bolus of 250 mL (Volulyte® 6%, Fresenius Kabi Deutschland GmbH, Bad Homburg, Germany, or Gelafundin ISO 40 mg mL^−1^, B. Braun Melsungen AG, Melsungen, Germany) was given within 5 min and repeated until reaching a SV plateau value (increase in SV < 10%). The optimum stroke volume (SV_opt_) was defined as the last successful fluid challenge, e.g. the last SV value just before reaching the plateau value, and SV_trigger_ as SV_opt_ minus 10%. Our GDT protocol is shown in Fig. 1. During surgery, our goal was to maintain SV above SV_trigger_ using fluid boluses, or inotropes (dobutamine or enoximone at 3 μg kg^−1^ h^−1^), when fluid loading was unable to restore SV values above SV_trigger_. Inotropes were not used in patients with two or more of the following conditions: existing coronary heart disease or angina pectoris, presence of diabetes mellitus, impaired renal function, or stroke in the patient’s history (Kristensen et al. 2014). Compliance to the haemodynamic treatment protocol was monitored using case report forms (CRFs) and evaluated postoperatively by two independent anaesthesiologists. Disagreement, if any, was solved by discussion with a third anaesthesiologist. The compliance rate was calculated as the number of protocol deviations divided by the total number of interventions during surgery and expressed as a percentage.

In the control group, haemodynamic management was left at the discretion of the attending anaesthesiologist.

### Outcome variables

The *primary outcome* measurement was the proportion of patients developing one or more postoperative complications during the hospital stay. All complications were extracted retrospectively from the electronic patient database management system by an independent medical documentation assistant, both for the GDFT and the control group using ICD-10-coded diagnoses in the medical records. The following postoperative complications were considered for analysis: infectious complications (wound infections, wound healing disturbances, pneumonia, urinary tract infections, sepsis, endocarditis, and peritonitis); cardiac complications (arrhythmias requiring medical treatment, pulmonary oedema, pulmonary embolism, myocardial infarction, and cardiovascular arrest); neurological complications (postoperative delirium and postoperative stroke); renal complications (increase of creatinine above twofold before surgery or need for dialysis); and haemorrhagic complications (postoperative bleeding with the need for postoperative red blood cell (RBC) transfusion).

*Secondary outcome* variables were postoperative need for vasopressors, postoperative complications, length of stay in the recovery room, post-anaesthesia care unit (PACU) or intensive care unit (ICU), the total length of hospital stay after surgery, and hospital mortality.

### Statistical analysis

Due to deviations from the normal distribution (Kolmogorov-Smirnov test), all analyses were performed non-parametrically. Results were expressed as median with 25th to 75th percentiles. Mann-Whitney *U* test and exact Fisher’s test were used for inter-group differences. Absolute and relative frequencies were used for categoric and dichotomous variables. Statistical analysis was carried out by using the Software Package for Social Sciences, 22.0 SPSS® for Macintosh (SPSS, Inc., Chicago, IL). A *p* value <0.05 was considered statistically significant.

The demographics and baseline covariates used for matching the GDFT group with an historical group were the age, the ASA score, and the P-POSSUM score, which are variables known to be independent predictors of postoperative morbidity and mortality. The ASA score and the P-POSSUM score were different between the groups when we analysed all the patients (GDFT group (*n* = 130) vs. control group (*n* = 258)) in favour of the control group. Several matching methods were excised in this study in order to find optimal balance using the identified baseline covariates. As a result, the individual matching method was chosen for this study and performed with the R package “optmatch” version 0.9-1.29 (Hansen and Klopfer 2006).

## Results

**Fig. 2:**
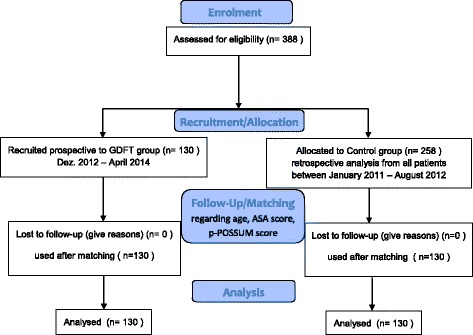
Flowchart recruitment HIPHOP

Between December 1, 2012, and April 30, 2014, 130 patients were recruited as part of the prospective GDFT group. Two hundred and fifty-eight patients underwent hip revision arthroplasty surgery between 1/2011 and 08/2012, and 130 were finally used for analysis and comparison after matching (Fig. 2).

The basic characteristics from patients (before matching) showed significant difference regarding the ASA score and the P-POSSUM score between the groups (Additional file 1: Table S1). The matching was performed using different parameters (age, ASA score and P-POSSUM score) to achieve equal distributions of the basic characteristics and the perioperative risk factors.

Before surgery, the matched patients from the GDFT group and from the control group were comparable (Table 1).

**Table 1:** Characteristics of the study population before surgery

	Control group (*n* = 130)	GDFT group (*n* = 130)	*p*
Age (years)	72 (60–76)	71 (62–75)	0.643
Sex (w/m)	86/44	81/49	0.440
Body height (cm)	166 (160–171)	168 (163–175)	0.155
Body weight (kg)	76 (65–85)	79 (64–90)	0.177
BMI kg/m^2^	27.36 (24.69–30.06)	27.77 (23.80–32.11)	0.658
CCS	3 (2–5)	3 (2–4)	0.249
ASA score	2 (2–3)	2 (2–3)	0.730
P-POSSUM score	27.00 (23.00–31.00)	29.00 (24.00–33.00)	0.102

Parameters are shown as median and (25th percentile–75th percentile). CCS: The Charlson Comorbidity Score includes age, previous myocardial infarction or congestive heart failure, peripheral vascular disease, cerebrovascular disease, existing dementia, COPD, connective tissue disease, peptic ulcer disease, diabetes mellitus, moderate to severe chronic kidney disease, hemiplegia, leukaemia, malignant lymphoma, solid tumour, liver disease, and AIDS. Different points were distributed for the pre-existing diseases, and so, the survival rate during the first 2 years can be calculated

During surgery, the GDFT protocol was properly followed 87.3% of the time. The overall fluid balance was comparable in both groups (Table 2). However, the GDFT group received more colloids and less crystalloids than the control group (Table 2). The GDFT group received more inotropes but not more vasopressors than the control group (Table 2).

**Table 2:** Intraoperative data of both groups

	Control group (*n* = 130)	GDFT group (*n* = 130)	*p*
Anaesthesia time (min)	185 (160–230)	197 (170–254)	0.056
Surgery time (min)	125 (99–159)	135 (107–171)	0.111
Total fluid (mL)	2210 (1658–3000)	2435 (1760–3480)	0.139
*Crystalloids (mL)*	*1500 (1000–2000)*	*725 (500–1000)*	*<0.001*
*Colloids (mL)*	*500 (500–1000)*	*1250 (1000–1750)*	*<0.001*
*Inotropes*	*1 [0.8]*	*28 [21.5]*	*<0.01*
Blood transfusion	47 [36.2]	57 [43.8]	0.255
NE at end of surgery	18 [13.8]	10 [7.7]	0.160
Admission recovery room	75 [57.7]	71 [54.6]	0.708
Admission PACU	44 [33.8]	53 [40.8]	0.305
Admission ICU	11 [8.5]	6 [4.6]	0.316

Parameters are shown as median (25th percentile–75th percentile) and number [percentage]

*NE* norepinephrine, *ICU* intensive care unit, *PACU* post-anaesthesia care unit

### Outcome variables

**Fig. 3:**
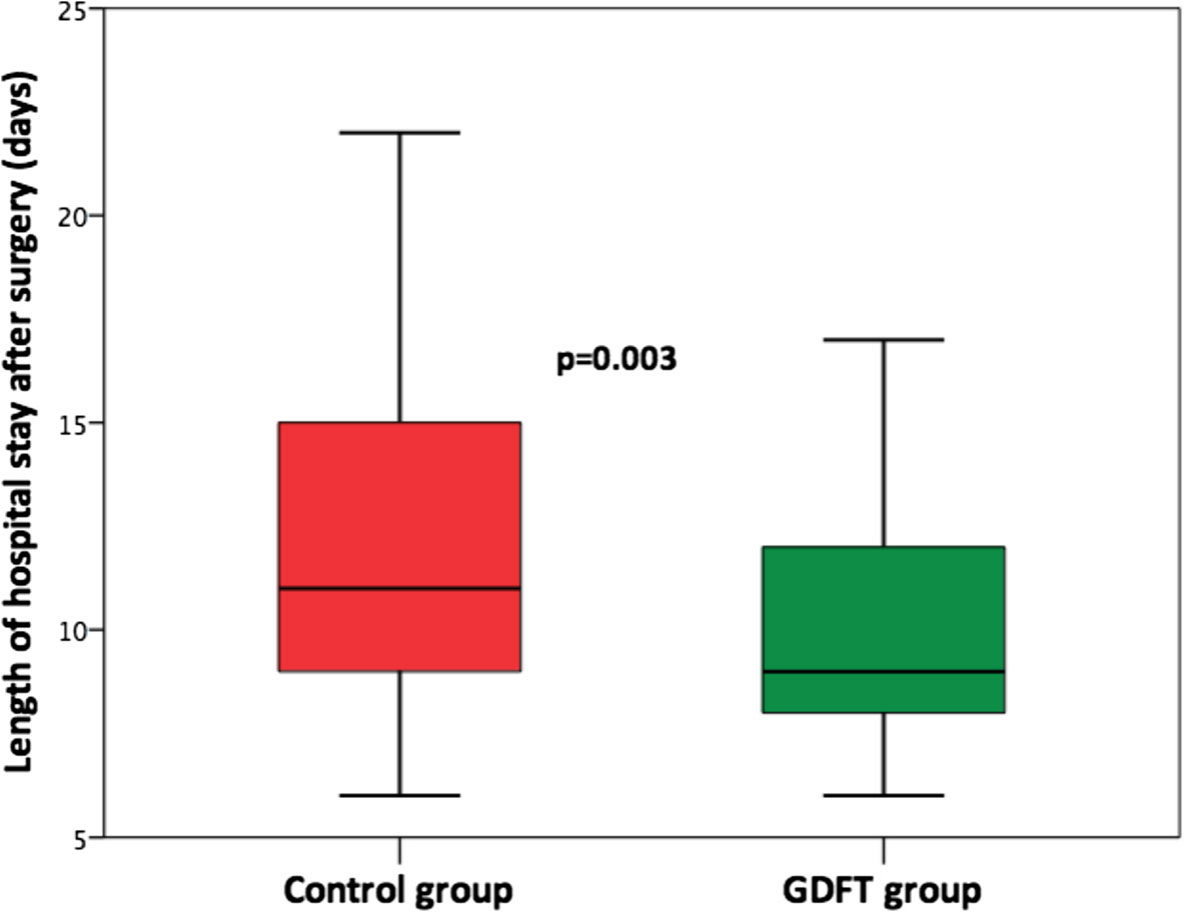
Postoperative hospital length of stay

**Fig. 4:**
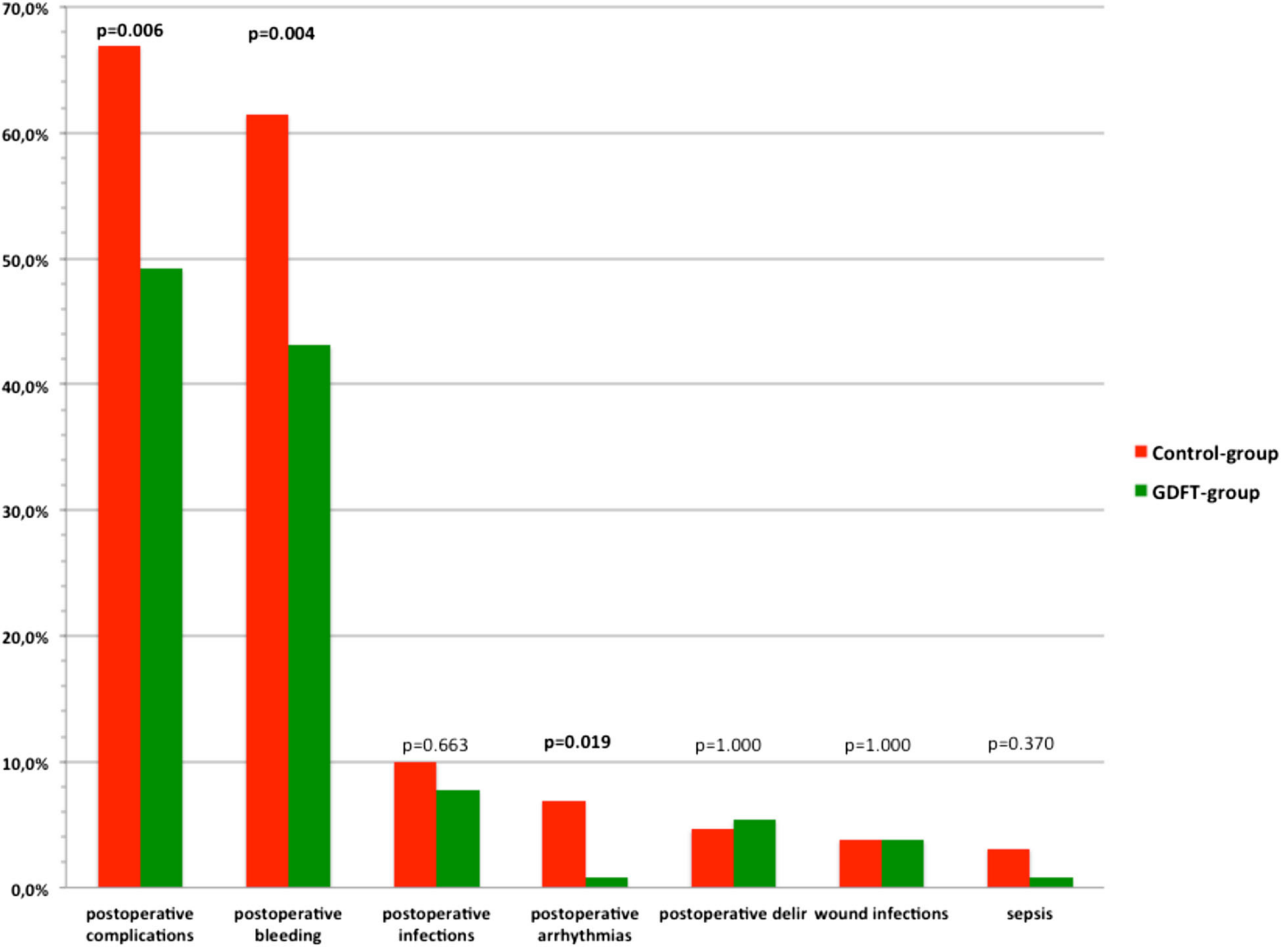
Postoperative complications

The postoperative morbidity rate was significantly lower in the GDFT group than in the control group (49.2 vs. 66.9%, *p* = 0.006) (Table 3). Postoperative LOS was significantly shorter in the GDFT group (11 days (9–15) vs. 9 days (8–12); *p* = 0.003) (Fig. 3). When patients were admitted in the ICU postoperatively, the patients from the control group stayed significantly longer in the ICU than the patients from the GDFT group (control group vs. GDFT group, 960 min (360–1210) vs. 400 min (207–825); *p* < 0.001). Other outcome variables are reported in Table 3 and Fig. 4.

**Table 3:** Total morbidity and complication rates

	Control group (*n* = 130)	GDFT group (*n* = 130)	*p*
*Total morbidity*	*87 [66.9%]*	*64 [49.2%]*	*0.006*
Infectious complications	13 [10%]	10 [7.7%]	0.663
*Cardiac complications*	*10 [7.7%]*	*2 [1.5%]*	*0.034*
*Postoperative arrhythmia*	*9 [6.9%]*	*1 [0.8%]*	*0.019*
Neurological complications	6 [4.6%]	7 [5.4%]	1.000
Renal complications	2 [1.5%]	2 [1.5%]	1.000
*Hemorrhagic complications*	*80 [61.5%]*	*56 [43.1%]*	*0.004*

Nevertheless, patients of the GDT group had significantly less postoperative cardiac complications (control group vs. GDT group, 7.7 vs. 1.5%; *p* = 0.034) (Table 3), especially as the new arrhythmias postoperatively occurred significantly more often in the control group (control group vs. GDT group, 6.9 vs. 0.8%; *p* = 0.019). The mortality rate was comparable between the groups (control group vs. GDT group, 0.8% (1) vs. 0.8% (1); 1.000).

## Discussion

In this matched cohort study and quality improvement project, we implemented into clinical routine a goal-directed fluid therapy protocol in patients undergoing revision hip surgery. The implementation was successful with a high protocol compliance rate of 87%. Patients from the GDFT had a significantly reduced postoperative morbidity (relative decrease of 26.5% (*p* = 0.006)), as well as a 2-day shorter length of hospital stay (*p* = 0.003) and also a shorter ICU stay (*p* < 0.001, when the patients were admitted to the ICU postoperatively).

Many studies have demonstrated the ability of GDFT to improve postoperative outcome in patients undergoing major abdominal and vascular procedures. Recent meta-analysis of randomized controlled trials (RCTs) suggests a 25–50% reduction in postoperative morbidity that was associated with a 1–2-day reduction in hospital length of stay (Grocott et al. 2012; Hamilton et al. 2011; Pearse et al. 2014). However, only few studies were done in patients undergoing orthopaedic surgery. In 1996, Sinclair et al. showed in 40 patients undergoing repair of proximal femoral fractures that fluid loading to an optimal stroke volume resulted in a more rapid postoperative recovery and a significantly reduced hospital stay (Sinclair et al. 1997). In 2002, Venn et al. showed that an invasive intraoperative haemodynamic monitoring concept using fluid challenges during repair of femoral fracture reduced the recovery time and also length of hospital stay (Venn et al. 2002). More recently, Cecconi et al. reported that in patients undergoing primary hip replacement under regional anaesthesia a goal-directed haemodynamic therapy changes the intraoperative fluid management and reduces postoperative complications (*p* = 0.05) (Cecconi et al. 2011). As far as we know, our study is the first investigating the effects of GDFT in patients undergoing more complex hip revision surgery.

In sharp contrast to this overwhelming evidence coming from RCTs, only little attention has been put on clinical implementation at the bedside and the value of GDFT in real life. Kuper et al. published in 2011 a multicentre trial where they implemented GDFT into clinical practice. In spite of a lower than expected (65%) adoption rate during the implementation phase, they observed a significantly shorter length of hospital stay with GDFT. Cannesson et al. recently published another real-life implementation programme of GDFT in patients undergoing major abdominal procedures and observed a 14% decrease in postoperative morbidity associated with decrease in median hospital length of stay from 10 (6–16) days to 7 (5–11) days (*p* = 0.0001) (Cannesson et al. 2015). In line with these publications, our study shows that implementation of GDFT is not only possible in a tertiary university medical centre but also might be beneficial for patients in whom the postoperative morbidity rate decreased by 26.5% and hospital length of stay decreased from 11 (9–15) days to 9 (8–12) days during the implementation phase. Our protocol was pretty simple and part of a newly created standard operating procedure officially approved by the departments of anaesthesiology and orthopaedic surgery. Both factors may have contributed to the high compliance rate and the clinical benefits we observed.

One point of discussion is the increased use of inotropes during surgery that might put the GDFT patients at an increased risk of myocardial ischemia and other cardiac complications. In contrast, the incidence of arrhythmia was significantly reduced in the GDFT group. The risk of myocardial ischemia and cardiac complications might have been mitigated by the fact that we used the risk assessment algorithm from the ESA/ESC to prevent patients being treated systematically with inotropes that are at high risk for myocardial ischemia (Kristensen et al. 2014). The adequate use of inotropes during surgery, based on SV data, may be helpful without increasing myocardial complications. This is in line with a recent meta-analysis which showed that the use of GDFT is associated with a decrease and not an increase in cardiac complications and in particular arrhythmias which are often triggered by hypovolemia (Arulkumaran et al. 2014). Other underlying factors that might have contributed to the reduced rate of cardiovascular complications might have been an improved microvascular perfusion leading to reduced systemic inflammation (Jhanji et al. 2010).

Another interesting finding was the reduced transfusion rate after surgery seen in the GDFT group. We did not change our transfusion guidelines during the study period, so it is unlikely that a change in transfusion practice could have explained it. It could be speculated that due to an improved intraoperative microcirculation in the optimized group, early bleeding during surgery is better recognized by the surgeon, and therefore, surgical haemostasis might be performed more effectively preventing later bleeding complications. Nevertheless, this is speculative and needs to be reproduced by further research.

*Our study has several limitations*. Given its before-after design, we cannot claim causality between the GDFT intervention introduced as a new standard of care and the observed changes in postoperative outcome. However, RCTs also have their limitations, particularly when blinding is not possible, as is the case when studying changes in clinical behaviour. Indeed, when performing a RCT where fluid management is standardized by the use of a predefined treatment protocol, clinicians are inevitably sensibilized and trained about the risk of giving too little or too much fluid during the perioperative period. As a result, they may change their usual practice and the so-called control group may not reflect anymore what used to be standard management in their institution. This “training effect” will tend to decrease the likelihood to show a difference between the intervention and the control group. On the other hand, when performing RCTs, clinicians usually benefit from extra human and financial resources, helping them to ensure the new strategy is properly implemented. This “resource effect” tends in contrast to increase the probability to show a difference between groups. In our study, the fluid management of the historical control group was not influenced at all by GDFT training and use since we introduced it only at the end of 2012. And when GDFT was introduced, it was used for all patients undergoing hip revision surgery as part of a new standard operating procedure. As a result, we believe our quality improvement study provides a pretty fair idea of the impact of GDFT implementation in real-life conditions and is complementary of previous RCTs done in orthopaedic patients, showing a benefit in more controlled conditions.

## Conclusions

In patients undergoing hip revision arthroplasty, the implementation of GDFT was associated with a significant decrease in postoperative morbidity and hospital length of stay. Our study confirms in real-life conditions what previous RCTs had suggested and is a clear invitation to expand our implementation to other surgical patient populations in whom postoperative complications remain an issue.

## Additional file

Additional file 1: Table S1. With basic characteristics of all patients before matching. **Table S2.** Intraoperative data of both groups. **Table S3.** Length of ICU stay. **Figure S1.** shows the postoperative hospital length of stay of all patients before matching. **Figure S2.** shows the postoperative complications of all patients before matching. (DOCX 62 kb)

## Abbreviations

GDFT: Goal-directed fluid therapy
ICU: Intensive care unit
PACU: Post-anaesthesia care unit
RBC: Red blood cells
SV: Stroke volume
SV_opt_: Optimal stroke volume
SV_trigger_: Trigger stroke volume
