# Supplementary material for: Implementation of goal-directed fluid therapy during hip revision arthroplasty: a matched cohort study
Source: Perioper Med (Lond). 2016 Dec 13;5:31. doi: 10.1186/s13741-016-0056-x (PMC5154150; doi:10.1186/s13741-016-0056-x)

Table S1: Basic characteristics of all patients before matching

|  | **Control-group (n=258)** | **GDFT-group (n=130)** | **p** |
| --- | --- | --- | --- |
| Age (years) | 71(60-77) | 71 (62-75) | 0.436 |
| Sex (w/m) | 155/103 | 81/49 | 0.741 |
| Body height (cm) | 168(160-174) | 168 (163-175) | 0.475 |
| Body weight (kg) | 76 (65-85) | 79 (64-90) | 0.244 |
| BMI kg/m^2^ | 27.34 (24.54-30.09) | 27.77 (23.80-32.11) | 0.554 |
| CCS | 3 (2-5) | 3 (2-4) | 0.059 |
| **ASA score** | **2 (2-3)** | **2 (2-3)** | **0.003** |
| **p-POSSUM score** | **25.00 (22.00-29.00)** | **29.00 (24.00-33.00)** | **<0.001** |

Table S2: Intraoperative data of both groups

|  | **Control-group (n=258)** | **GDFT-group (n=130)** | **p** |
| --- | --- | --- | --- |
| **Anaesthesia time (min)** | **176 (150-220)** | **197 (170-254)** | **<0.001** |
| **Surgery time (min)** | **116 (95-145)** | **135 (107-171)** | **<0.001** |
| **Total fluid (mL)** | **2000 (1500-2720)** | **2435 (1760-3480)** | **<0.001** |
| **Crystalloids (mL)** | **1500 (1000-2000)** | **725 (500-1000)** | **<0.001** |
| **Colloids (mL)** | **500 (500-1000)** | **1250 (1000-1750)** | **<0.001** |

| **Inotropes** | | **4 [1.6]** | **28 [21.5]** | **<0.001** |
| --- | --- | --- | --- | --- |
| **Blood transfusion** | | **62 [24.0]** | **57 [43.8]** | **<0.001** |
| NE at end of surgery | | 24 [9.3] | 10 [7.7] | 0.705 |
| **Admission recovery room** | | **173 [67.1]** | **71 [54.6]** | **0.019** |
| **Admission PACU** | | **70 [27.1]** | **53 [40.8]** | **0.008** |
| Admission ICU |  | 16 [6.2] | 6 [4.6] | 0.645 |

NE - Norepinephrine, ICU – intensive care unit, PACU – post anaesthesia care unit

Parameter are shown as median (25^th^ percentile – 75^th^ percentile) and number [percentage].

Table S3: postoperative stay on ICU

|  | **Control-group (n=258)** | **GDFT-group (n=130)** | **p** |
| --- | --- | --- | --- |
| **Length of ICU stay (min)** | **617 (360-1115)** | **400 (207-825)** | **<0.001** |

Figure S1: Postoperative hospital length of stay of all patients before matching


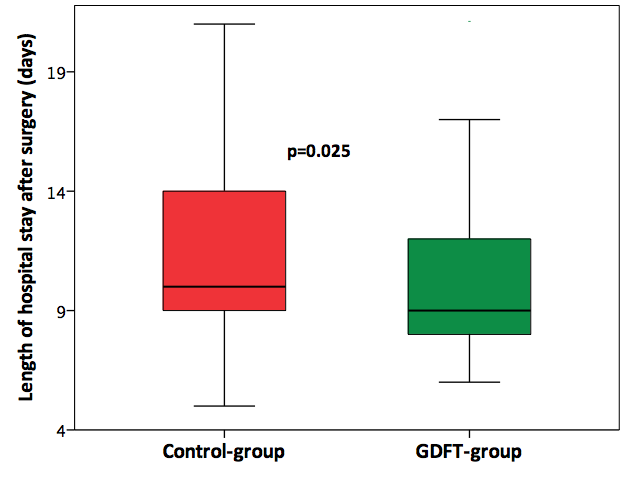


Figure S2: Postoperative complications


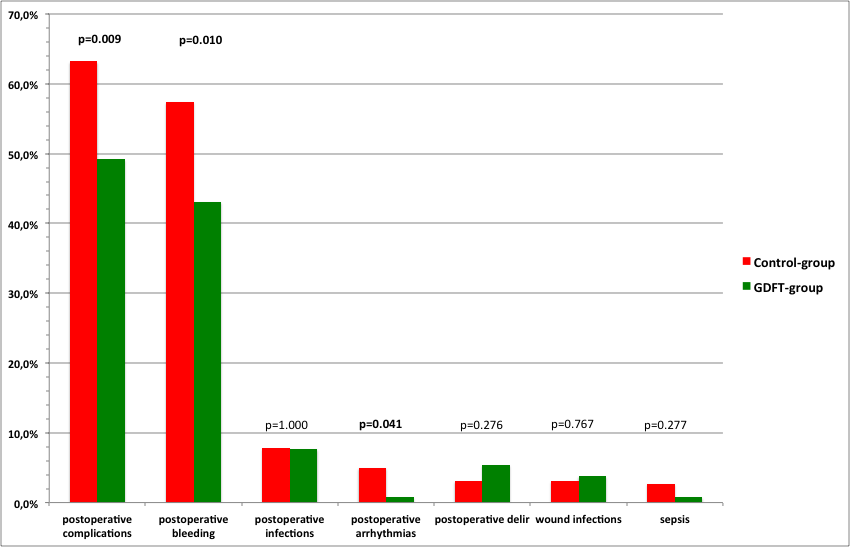

Supplement: Additional file 1: Table S1. — With basic characteristics of all patients before matching. Table S2. Intraoperative data of both groups. Table S3. Length of ICU stay. Figure S1. shows the postoperative hospital length of stay of all patients before matching. Figure S2. shows the postoperative complications of all patients before matching. (DOCX 62 kb) [file 13741_2016_56_MOESM1_ESM.docx]
